# Supplementary material for: GmSWEET29 and Paralog GmSWEET34 Are Differentially Expressed between Soybeans Grown in Eastern and Western Canada
Source: Plants (Basel). 2022 Sep 7;11(18):2337. doi: 10.3390/plants11182337 (PMC9502396; doi:10.3390/plants11182337)
Supplement: Supplementary file 1 [file plants-11-02337-s001.zip › Hooker2022_Supplementary-Figures_with_captions_v2.pdf]

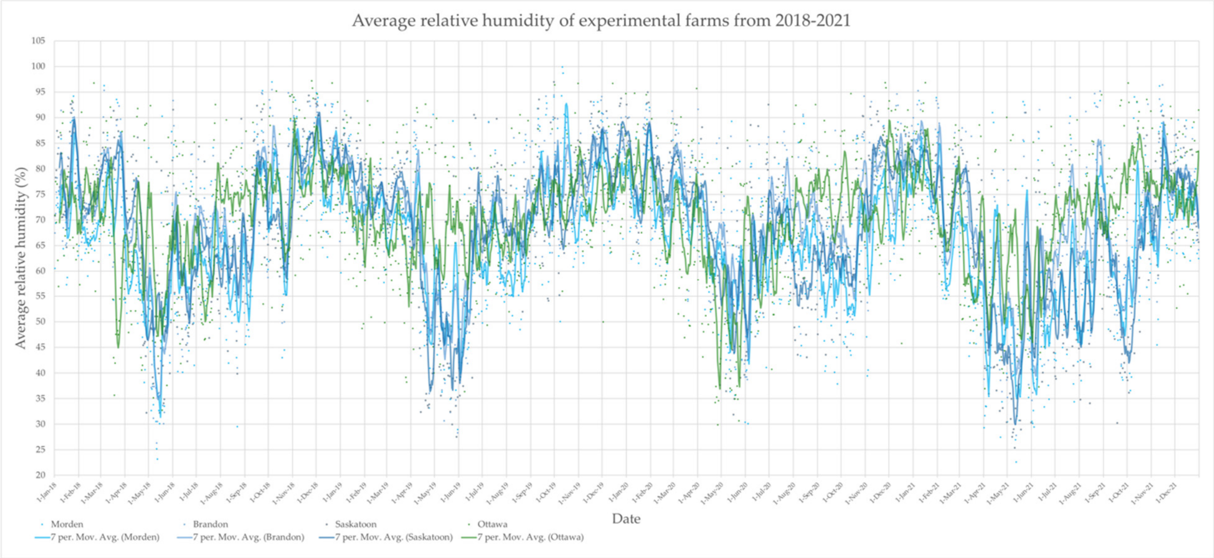

Supplementary Figure S1: Average relative humidity (%) of experimental farms from 2018-2021. East (Ottawa) data is represented in green, West (Morden, Brandon, Saskatoon) data is represented in shades of blue. Daily relative humidity data are represented by individual data points and the 7-day moving average is represented by solid lines.

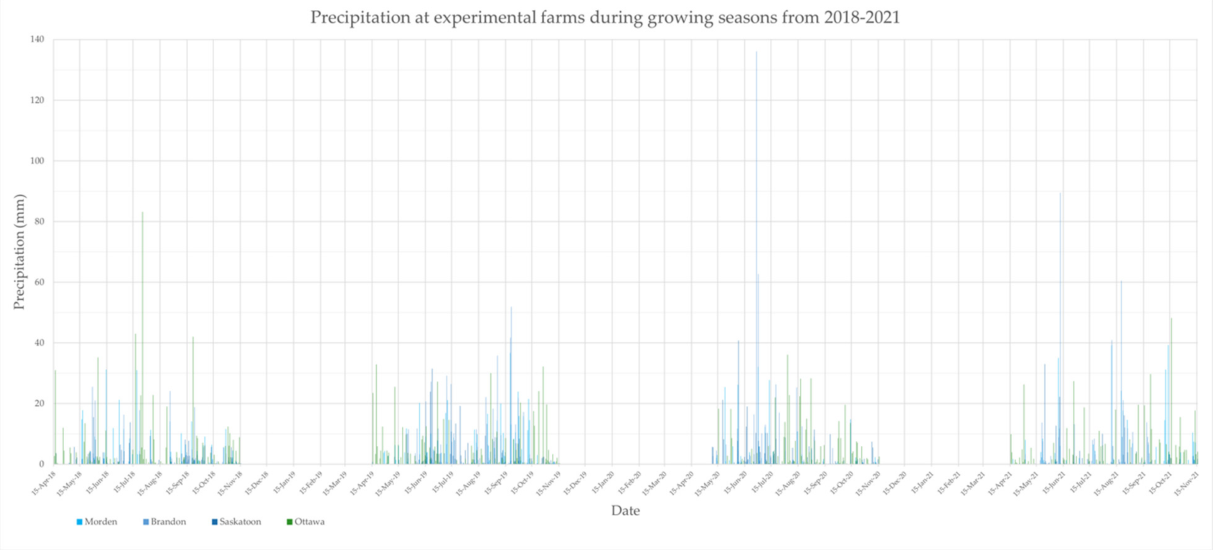

Supplementary Figure S2: Total precipitation (mm) during growing seasons (mid-March to mid-November) for each experimental farm from 2018-2021. East (Ottawa) data is represented in green, West (Morden, Brandon, Saskatoon) data is represented in shades of blue.

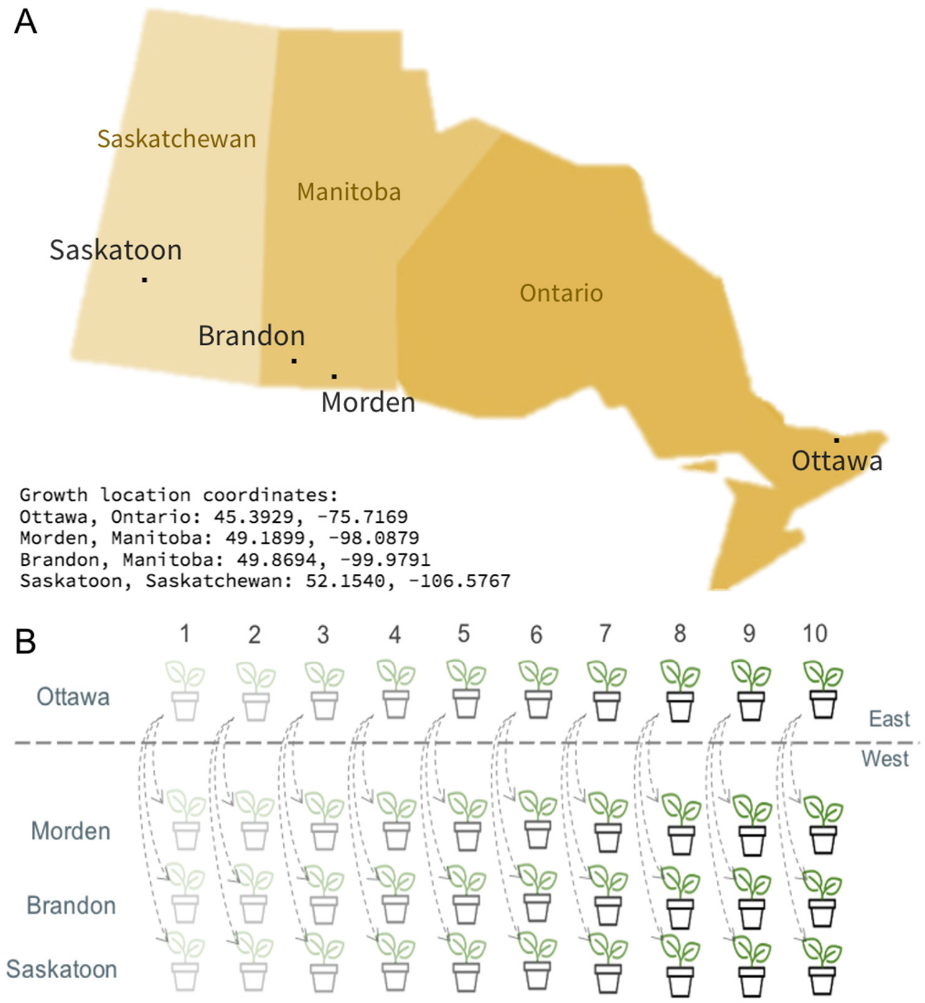

Supplementary Figure S3A
Map of planting locations in Canada with coordinates; Ottawa Ontario, Morden Manitoba, Brandon Manitoba, and Saskatoon Saskatchewan. Ottawa represents the eastern growing regions (control) and Morden, Brandon, and Saskatoon represent the western growing regions (experimental). Ten lines ranging in seed protein content have been planted in East and West growing regions from 2018-2021.

Supplementary Figure S3B
Differential expression pairwise comparisons between East and West for lines 1-10. Leaf tissue was sampled in triplicate at the R5 stage and assessed for RNA expression and DE analysis between East and West. Ottawa (control) represents the East, Morden, Brandon and Saskatoon (experimental) represent the West. Pairwise comparisons (represented by dashed arrows) were only made between identical genotypes with Ottawa always serving as the control. Planting and sampling took place over 2018 (Morden, Brandon), 2019 (Morden, Brandon, Saskatoon), 2020 (Morden, Saskatoon), and 2021 (Morden, Brandon, Saskatoon; exclusive of Line 1).
