# Supplementary figures and images for: GmSWEET29 and Paralog GmSWEET34 Are Differentially Expressed between Soybeans Grown in Eastern and Western Canada
Source: Plants (Basel). 2022 Sep 7;11(18):2337. doi: 10.3390/plants11182337 (PMC9502396; doi:10.3390/plants11182337)

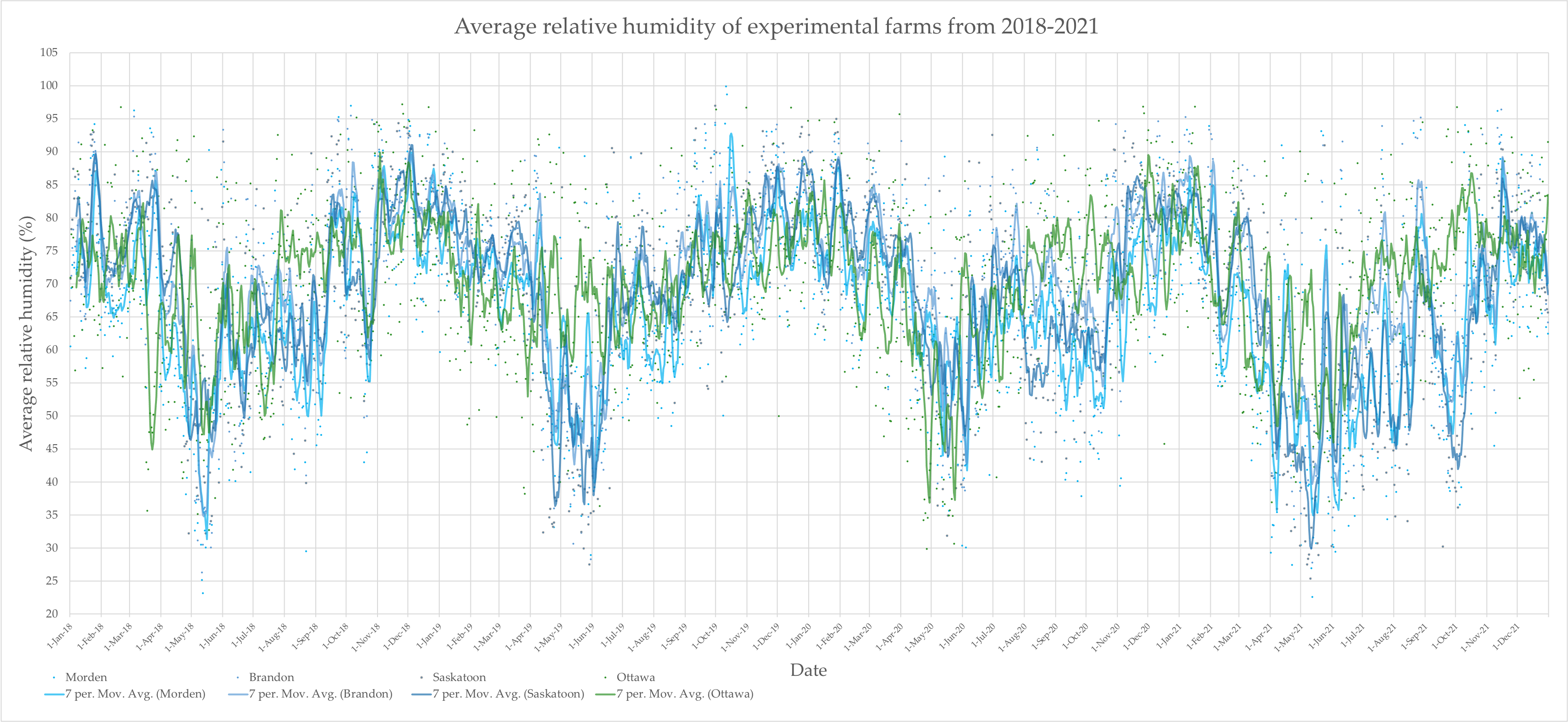

Supplement: Supplementary file 1 [file plants-11-02337-s001.zip › Hooker2022_manuscript_SF1.png]

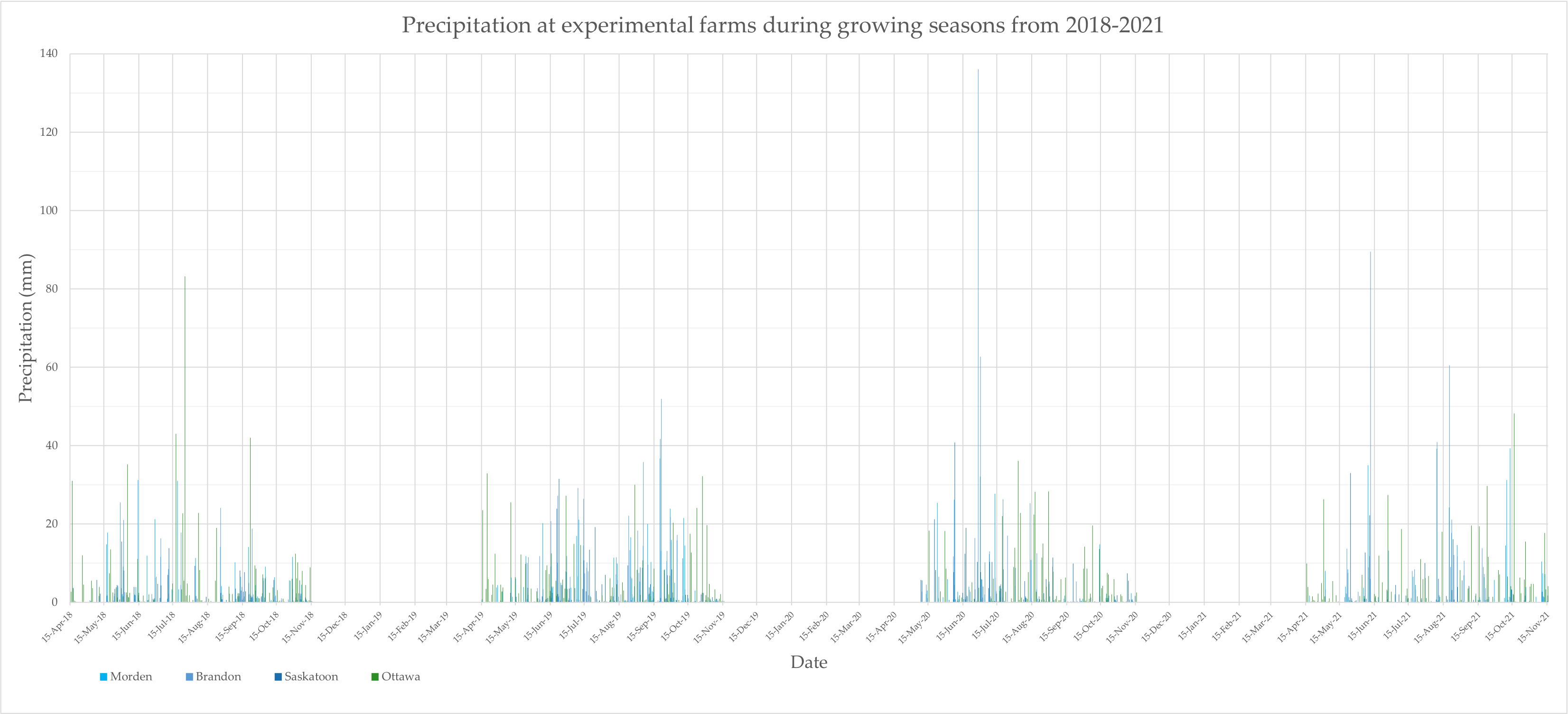

Supplement: Supplementary file 1 [file plants-11-02337-s001.zip › Hooker2022_manuscript_SF2.png]

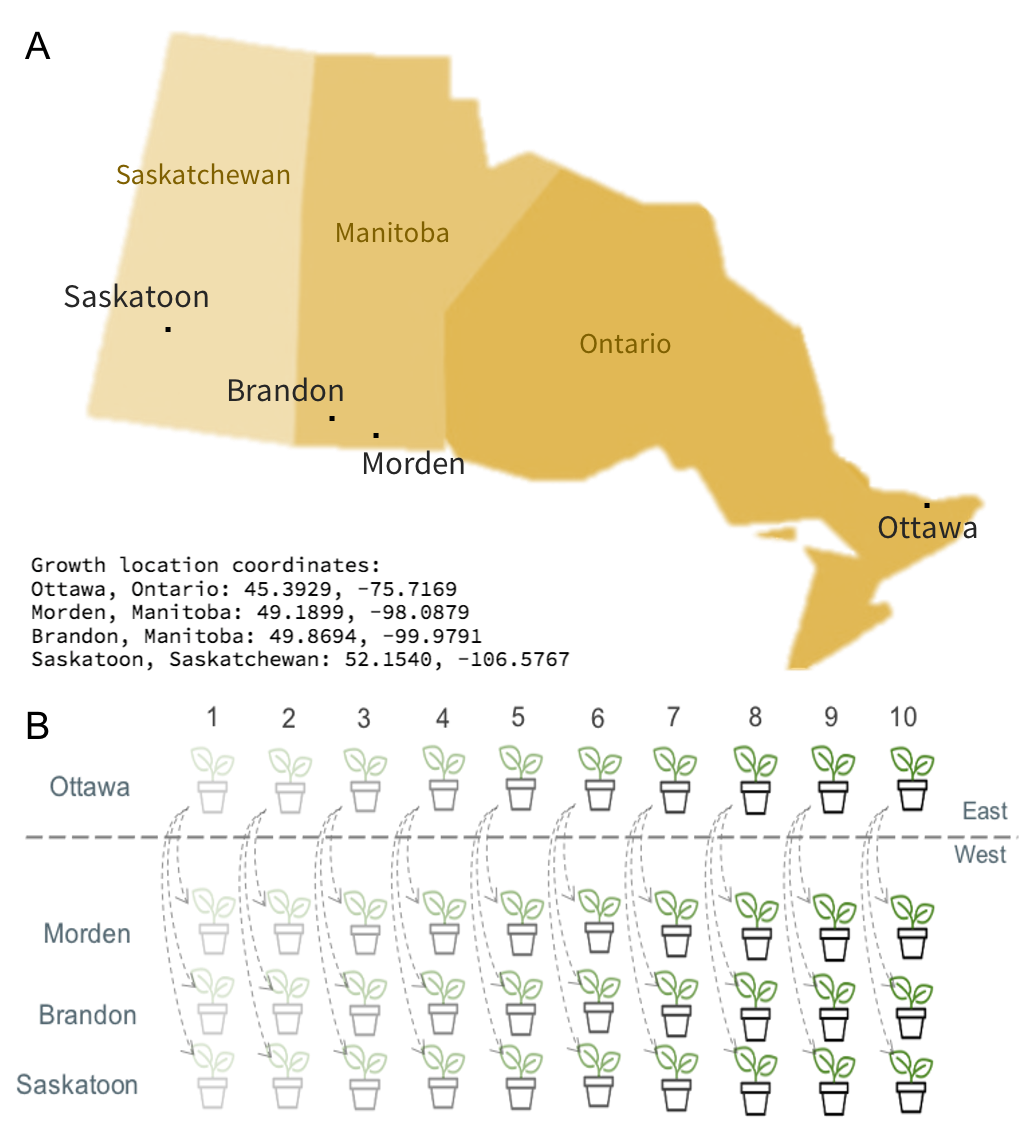

Supplement: Supplementary file 1 [file plants-11-02337-s001.zip › Hooker2022_manuscript_SF3.png]
